# Need Assessment for Specialised Hospitals at the Regional Level: A Potential Way Out of Medical Tourism

**DOI:** 10.7759/cureus.105491

**Published:** 2026-03-19

**Authors:** Md Ferdous Ahmed, Prabir Bhowmik, Mohammad Asif Khan

**Affiliations:** 1 Civil Engineering, Military Institute of Science and Technology (MIST), Dhaka, BGD; 2 Public Administration, Chittagong University of Engineering and Technology (CUET), Chattogram, BGD; 3 Program Development for Mental and Primary Health, Base Care Foundation, London, GBR

**Keywords:** healthcare at regional level, health tourism, medical tourism, need assessment for treatment care, specialised medical care

## Abstract

Background

Every year, a significant number of people go abroad to access healthcare, specifically to access medical care for life-threatening clinical conditions and diverse chronic conditions such as cardiovascular diseases, respiratory diseases, renal diseases, and cancer for them and their families, due to a lack of access to specialised medical care within their reach and a lack of compliance with the existing healthcare facilities.

Methodology

This cross-sectional study was conducted to evaluate the morbidity profile of respondents and their families in relation to their treatment-seeking behaviour abroad and the reasons for it using a pre-tested questionnaire. Along with the descriptive analysis, further analysis was performed by utilising principal component analysis to determine the need for specialised treatment centres at the regional level of the country, as perceived by the respondents, as a means for reducing the ongoing trend of medical tourism.

Results

In the observed group (n = 523), 26.19% went abroad to seek medical care, while more than half of the respondents (54.49%) expressed their intention to go out of the country for their treatment purposes. Collectively, an overwhelming 90.82% of the respondents desired specialised hospitals at the regional level, emphasising the need for healthcare facilities dedicated to cardiovascular diseases, respiratory diseases, renal diseases, diabetes, and cancer at the regional level.

Conclusions

Establishment of specialised hospitals at the regional level in the country, considering the prevailing health conditions, may reduce the trend of medical tourism by fellow countrymen, mitigating the need for accessible, quality medical care and opening the scope for health tourism by people in neighbouring countries.

## Introduction

Bangladesh is a densely populated country, being the eighth most populous country in the world, with 2.3% of the world’s population [1]. Bangladesh has a substantial medical workforce of 141,999 registered physicians (MBBS and BDS) and 0.83 doctors per 1,000 population, which is far behind the World Health Organization (WHO) standard, and 9.9 health workers per 1,000 population, compared to the international standard of 44.5 [2], highlighting a shortage of health professionals. The country is being served by 71,100 total hospital beds at government hospitals (2,405 population/hospital bed) through 37 government medical college hospitals, six armed forces medical college hospitals, 62 district and general hospitals, 429 Upazilla health complexes, and 17 tertiary-level specialised hospitals [2]. As a result, in Bangladesh, hospitals often admit more patients than their bed capacity, leading to compromised healthcare delivery.

Bangladesh has historically been a hotbed for various infectious diseases. However, with socioeconomic development, the types of disease are changing. Although infectious diseases are still prevalent, non-communicable diseases (NCDs) currently dominate the disease profile, contributing to the major causes of morbidity and mortality. It was estimated that NCDs caused 74% of all deaths in Bangladesh (compared to 70.26% globally), with 34% of deaths due to cardiovascular disease, 14% due to cancer, 11% due to other NCDs, and 4% due to diabetes [2].

For curative services for different infectious diseases, NCDs, and life-threatening conditions, most people depend on government hospitals, especially on medical college hospitals and specialised hospitals. Despite a moderate number of private and non-government organisation (NGO) health facilities, including 73 private medical college hospitals, 4,172 private hospitals and clinics, and 1,610 NGO hospitals and clinics across the country [2], people prefer government facilities for major health conditions and emergencies such as cardiovascular diseases, chronic respiratory disease, cancer, chronic kidney diseases, respiratory diseases, and diabetes, as most of the private and NGO hospitals are delivering primary and secondary levels of medical care, with very few offering specialised treatment facilities.

However, as most specialised healthcare facilities are in Dhaka, the capital city (13 out of 17), people are interested in seeking treatment abroad, considering the comparative benefit provided by the advanced treatment facilities, readily accessible specialised care, enhanced compliance with the healthcare delivery system, and perceived reliability of the diagnostic procedures and skill levels of healthcare professionals in foreign facilities [3]. Besides advanced medical care facilities, geographical proximity, ease of barriers in reaching the destination, familiarity, and cultural similarity function as significant determinants of the destination country.

People go abroad for various treatment purposes, among them cardiovascular diseases are at the top, accounting for 17% of the total patients, followed by 14.5% for renal conditions, 11.5% for orthopedic surgeries, 11% for liver diseases and cancer, 6% for gastroenterology and urology, 5% for ear, nose, and throat conditions, 4% for performing general surgeries and gynecological interventions, and 2% for treating eye and dental conditions, with more than 2,700,000 people going abroad every year for treatment in more than 19 countries, including India, Singapore, Thailand, and Malaysia. Most go to India (92%) for seeking medical care, causing an estimated yearly 45,000,000 dollars of valuable foreign currency to go out of the national economy [4].

Service quality, word-of-mouth communication, personal recommendations, and positive reviews from friends, family, and the internet significantly affect outbound medical tourism, with perceived quality of healthcare facilities having an influential effect. Though researchers from developed countries and different disciplines have conducted empirical studies on medical tourism, little is known about this phenomenon in developing countries [5]. In that regard, based on the experiences from countries renowned for medical tourism, Bangladesh needs to take holistic initiatives, including continuous technical and behavioral development of health professionals, improving existing hospital management and diagnostic efficiency, developing favorable health policy, and establishing specialised healthcare facilities at the regional level throughout the country within the reach of the people to prevent outbound medical travel [6,7].

Despite the resource constraints, over the last few decades, Bangladesh has made remarkable progress in achieving both health and population indicators such as Millennium Development Goals (MDGs) and noticeable progress toward achieving Sustainable Development Goals (SDGs) [8], which is immensely instrumental in ensuring the rights to health and exemplary success in reducing maternal and child mortality and vaccination coverage [9]. However, the healthcare sector in Bangladesh is still suffering from several problems that pose challenges to establishing a rights-based and service-oriented healthcare sector due to flawed regulatory frameworks, forfeiting accountability and transparency, widespread corruption, a poor monitoring system, inadequate health financing, and inequity between the rural and urban populations in accessing healthcare services [10]. While the trend of seeking medical treatment abroad highlights the existing gaps in the Bangladesh healthcare system, it also underscores the need for systemic improvements. Addressing these challenges through targeted investments, policy reforms, and international collaborations can enhance the quality and accessibility of healthcare in Bangladesh, potentially reducing the need for citizens to seek treatment abroad [11].

Considering the context of the existing trend in medical tourism in the country, this study was designed to evaluate the following: (1) morbidity profiles of individuals and their family members leading to health-seeking behaviours in neighbouring countries; (2) reasons for choosing foreign medical facilities over home for understanding the context of the need assessment for the establishment of specialised medical care facilities at the regional level; and (3) evaluating the division-wise disease prevalence and subjective understanding of the reported gaps in accessible and affordable treatment facilities to sort out a way to bring about a significant reduction in medical tourism by Bangladeshis.

## Materials and methods

This study was conducted using a Google Forms questionnaire disseminated via social media platforms (in various health-related Facebook groups) from January 2025 to March 2025 to include individuals active on social media platforms, presumably in groups using online sources to choose their destinations for medical tourism. The questionnaire was initially developed by interviewing individuals conveniently known to the researchers, updated based on an extensive literature review, and finally pretested in a relevant cohort. A total of 617 responses were collected, but 523 were considered in the analysis due to incomplete responses. During the collection and analysis of the data, confidentiality and anonymity were strictly maintained. The study obtained approval from the Ethical Review Board of the Military Institute of Science and Technology (MIST), Bangladesh (approval number: MIST/IRB/2025/1).

Demographic and morbidity profile

A descriptive analysis of demographic variables and morbidity profiles of the respondents and their family members and division-wise demand for specialised hospitals was performed using SPSS Statistics version 31 (IBM Corp., Armonk, NY, USA).

DBSCAN clustering

The machine learning component of the analysis utilised the Density-Based Spatial Clustering of Applications With Noise (DBSCAN) algorithm to identify natural groupings within the survey data, based on respondents’ demographic and healthcare preference features. Before clustering, age, occupation, division, medication usage, foreign treatment history, and need for specialised hospitals were pre-processed. Categorical features were label-encoded, and all features were standardised to ensure comparability.

DBSCAN was applied with an epsilon parameter of 1.2 and a minimum sample size of 5 to detect clusters of similar respondents while identifying noise points as outliers. The resulting clusters were visualised using principal component analysis (PCA) to reduce dimensionality to two components. This approach facilitated the interpretation of complex multivariate relationships within the data and provided insight into distinct patient profiles related to medical treatment preferences.

## Results

Demographic profile

The age range of the respondents was 20-75 years (mean ± SD = 35.02 ± 10.55 years), with a higher participation by males (54.9%) compared to females (45.1%). Almost half of the respondents were from Chittagong (48%), followed by Rajshahi (14.9%) and Dhaka (10.3%). Most of the participants were educated up to graduation level (42.1%), which contributed to a better quality of response in terms of understanding the questionnaire and providing insightful opinions on the further need for specialised facilities. There was an overwhelming response from service holders (61.8%) and noticeable responses from individuals belonging to the upper socioeconomic class (53.7%) (Table 1).

**Table 1:** Demographic profile of the respondents (n = 523).

Variable	Frequency	Percentage
Sex		
Male	287	54.9%
Female	236	45.1%
Division		
Chattogram	251	48%
Dhaka	54	10.3%
Sylhet	32	6.1%
Mymensingh	20	3.8%
Khulna	26	5%
Rajshahi	78	14.9%
Rangpur	33	6.3%
Barishal	29	5.5%
Educational status		
Up to primary	1	0.2%
Up to secondary	73	14%
Up to higher secondary	156	29.8%
Up to graduation	220	42.1%
Postgraduation	73	14%
Socioeconomic status		
Low	53	10.1%
Middle	189	36.1%
Upper	281	53.7%
Occupation		
Service holder	323	61.8%
Housewife	6	1.1%
Student	36	6.9%
Business	23	4.4%
Others	135	25.8%

Morbidity profile

The specific morbidity profile of the 247 respondents and their family members primarily included non-communicable, chronic diseases such as diabetes, hypertension, and diseases of the cardiovascular, renal, hepatobiliary, and respiratory systems, including cancers and strokes. Frequencies of morbidities showed a similar pattern in both groups, with diabetes and hypertension being the most frequent isolated diseases, as evidenced by 8.5% of the participants and 11.3% of the family members having diabetes and 12.6% of the participants and 8.9% of the family members suffering from hypertension (Table 2). There was an alarming rate of comorbidities among the respondents, with 22.2% having three or more comorbidities, and even higher levels of comorbidities in their family members, with 40.5% having three or more comorbidities, as shown in Table 2.

**Table 2:** Morbidity profile of the respondents and their family members (n = 247).

Disease	Respondents (n = 247)		Family members (n = 247)	
	Frequency	Percentage	Frequency	Percentage
Diabetes	21	8.50%	28	11.33%
Hypertension	31	12.55%	22	8.91%
Renal diseases	1	0.40%	0	0
Cardiovascular diseases	6	2.42%	9	3.64%
Respiratory diseases	16	6.47%	11	4.45%
Hepatobiliary diseases	6	2.42%	4	1.62%
Cancer	1	0.40%	0	0
Others	51	20.66%	30	12.15%
Two comorbidities	59	23.90%	43	17.41%
Three comorbidities	26	10.53%	55	22.27%
More than three comorbidities	29	11.75%	45	18.22%

Reasons for health tourism

**Figure 1:**
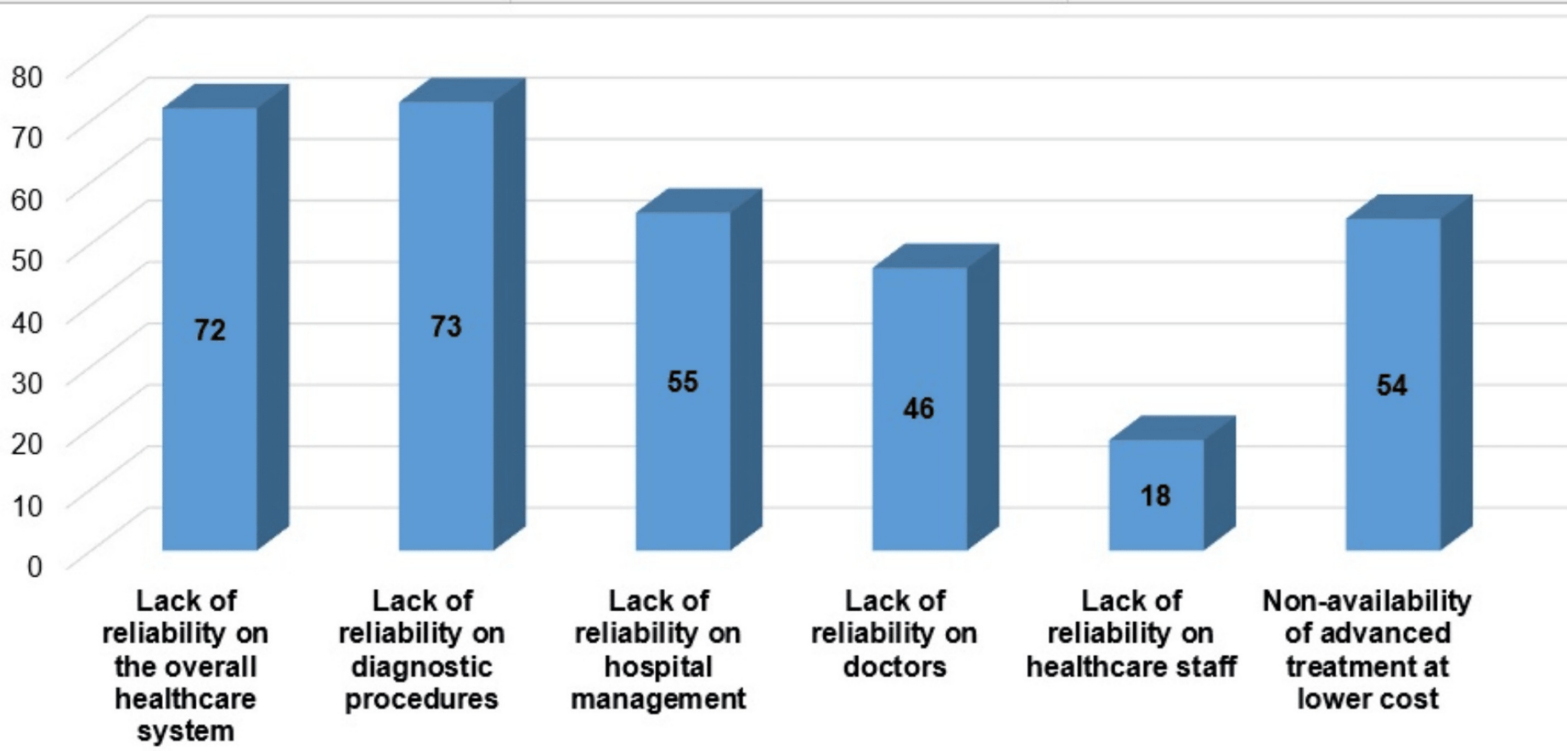
Reasons for seeking treatment abroad (n = 137). *: There were multiple responses by the respondents.

Out of the reasons for seeking medical care out of the country, lack of reliability on the diagnostic procedures was the highest (n = 73), followed by lack of reliability on the overall healthcare system (n = 72) and lack of reliability on doctors (n = 55) (Figure 1).

Willingness to go abroad

**Figure 2:**
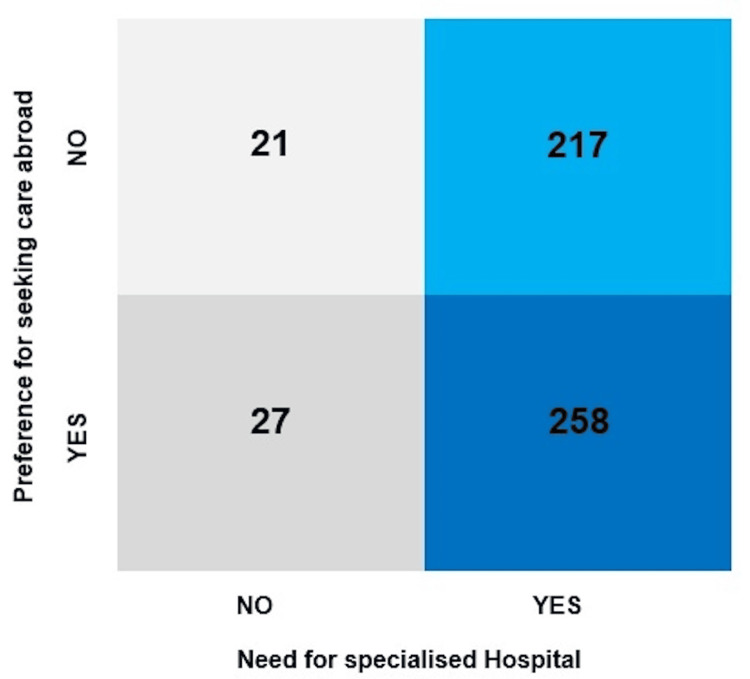
Relationship between willingness to seek treatment abroad and need for specialised hospital (n = 523).

A noticeable number of individuals intending to go abroad for medical care expressed the need for specialised hospitals (258) (Figure 2).

Division-wise need for specialised hospitals

According to the concerns shared by the respondents, there was a higher demand for specialised hospitals for cardiovascular disease, followed by diabetes, cancer, and renal diseases across the divisions (Table 3).

**Table 3:** Division-wise need for disease specific specialised hospitals (n = 523).

Division	Need-based type of hospital							
	Cancer	Cardiovascular diseases	Diabetes	Hepatobiliary diseases	Hypertension	Others	Renal diseases	Respiratory diseases
Barishal	13	20	18	13	18	7	12	12
Chattogram	144	192	145	118	104	68	98	111
Dhaka	28	41	37	24	28	11	19	28
Khulna	13	24	20	15	17	6	17	15
Mymensingh	11	9	10	5	10	8	9	8
Rajshahi	27	36	49	24	31	34	26	42
Rangpur	26	17	18	12	11	8	14	11
Sylhet	22	24	15	11	14	6	12	15

DBSCAN clustering map

**Figure 3:**
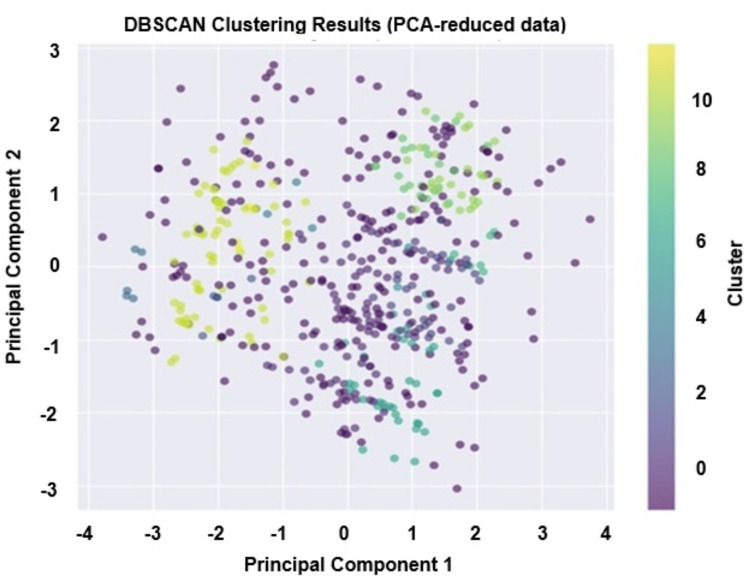
DBSCAN clustering map identifying distinct groups within the survey respondents based on demographic features (n = 523). DBSCAN: Density-Based Spatial Clustering of Applications With Noise; PCA: principal component analysis

The DBSCAN clustering analysis (Figure 3) identified multiple distinct groups of the survey respondents, revealing varied patterns of medical treatment preferences and demographic features. The noise points were characterised by a moderate average age of 35 years, a foreign treatment preference rate of 55.70%, and a high need for specialised hospitals at 79.70%.

## Discussion

Medical tourism is a form of travel aimed at receiving medical treatment with hospital visits and special arrangements such as specific treatment interventions, ambulance services, and supportive medical care in foreign countries. This observational study was conducted to explore the need for specialised hospitals at the regional level of Bangladesh dedicated to the prevailing health conditions at the community level as a way out for outbound medical tourism. It also considered the dimension of health tourism caused by a lack of access to appropriate healthcare services for diverse health conditions at the divisional level, as specialised hospitals are aggregated in the capital city of Dhaka. The study provided significant insights into the need assessment for tertiary-level hospitals outside the capital, which could cause a reduction in seeking medical treatment abroad.

Demographic profile

Participation in the study was by individuals with a wider age range, from 20 years to 75 years, covering all the age groups, including younger, middle-aged, and elderly, with a slightly higher response from males (54.9%) (Table 1). Understandably, the disease profile varied considerably in children and adolescents compared to the age group covered by this study. However, the scope for tertiary-level medical care for children and adolescents was beyond the scope of this study.

Among the participants, most hailed from Chattogram division (251 out of 523), followed by Rajshahi (n = 78), and Dhaka (n = 54), while the minimum participation was observed from Mymensingh (n = 20), followed by Khulna (n = 26), Barishal (n = 29), Sylhet (n = 32), and Rangpur (n = 33) (Table 1), being the smaller division in terms of population in relation to the divisions with higher population (i.e., 25.4% of the total population of Bangladesh live in Dhaka division while 5.8% of the total population live in Sylhet division) [12]. This distribution reflected a closer representation of division-wise inhabitation, other than the highest participation from the Chattogram division, as Dhaka is the most populous division of the country.

There were participants from different educational status ranging from up to primary to postgraduation level of study, with the highest number of respondents educated up to the graduation level (42.1%), followed by those educated up to higher secondary (29.8%), and equally by education up to secondary and postgraduation level (14%) (Table 1), probably explained by the increased willingness of the people educated at the graduate level to contribute to the study purpose and better understanding of the context of the study. Inevitably, a significant proportion of the population was not included in the study, who had a lower level of education due to their limited access to social media platforms and the capacity to participate in online surveys. A noticeable number of males (23%) and females (24%) had no education, and the net attendance ratios for primary school and secondary school are 68% and 48%, respectively, in the country [12].

Economically, most of the respondents belonged to the upper social class (53.7%), followed by the middle class (36.10%), and the lower socioeconomic class (10.1%). In our consideration, it was partly contributed by participations from urban habitants and partly by the fact that the formal monthly family income level considered for classifying the socioeconomic status was according to the conventional consensus (monthly family income less than 12,500 BDT for lower, 12,500 to 21,500 for middle, and more than 21,500 for upper group) [13] and might not be the real expression of the wealth of the individuals, as the criteria for identification of socioeconomic status based on national median income works differently for developing countries, with the determination of socioeconomic class being influenced by factors beyond family income [13].

We noticed overwhelming participation by service holders (61.8%), followed by other categories of occupation (25.8%), including self-employment and small-scale economic activities (Table 1). However, individuals from an important type of occupation involved in agricultural activities were not reported, which might be due to the lack of access to social media platforms.

Morbidity profile

Understanding the morbidity profile at the regional level is pivotal for effective planning and decision-making regarding the establishment of a dedicated specialised hospital, as it provides a specific outline of the disease pattern in each locality, causing people to spend on medical tourism in search of appropriate treatment care. We considered evaluating the morbidity profile for both groups, as we were aware that either the conditions affecting the individuals or family members contributed to their treatment plan.

In both the respondents and family members, prevailing isolated conditions included diabetes, hypertension, cardiovascular diseases, respiratory diseases, hepatobiliary diseases, cancer, and renal diseases (Table 2), which were among the top 10 reported causes of hospital admissions and top 20 registered certified causes of deaths nationwide [2]. We found a higher trend of comorbidities for both groups, extending up to 11.75% of respondents and a staggering 18.22% of family members having more than three conditions (n = 247) (Table 2), followed by 23.90% of participants and 17.41% of family members suffering from two comorbidities, and 10.53% and 22.27% of the participants and family members, respectively, suffering from three comorbidities (Table 2), with conditions such as diabetes, hypertension, and respiratory disease being the frequently observed comorbidities. This morbidity outlook provided useful insight into the fact that, considering the initiation of specialised hospitals, the focus needs to be on dealing with multiple prevalent health conditions rather than focusing on an isolated disease.

There was also a noticeable presence of other diseases, including infectious diseases, i.e., malaria, typhoid, fever etc.; neglected tropical diseases, i.e., filariasis, helminthiasis etc.; and other metabolic diseases, i.e., thyroid disorders, observed with an increased frequency among the respondents (20.66%), while 12.15% of the family members were suffering from similar conditions (Table 2). These findings helped in understanding that though Bangladesh has been successful in dealing with a diverse group of infectious diseases over the last few decades and facing an ever-growing number of NCDs at the community level, the efforts need to be ongoing for dealing with different communicable diseases and the neglected tropical diseases.

Reasons for medical tourism

We aimed to determine the reasons for medical tourism as experienced by the individuals seeking medical care abroad (n = 137) either for them or their family members to highlight the causes of seeking treatment abroad, as these concerns are necessary to address for designing the operational outline of specialised hospitals at the division level for a satisfactory service provision and wider accessibility of healthcare facilities by the local community. The findings showed that there was a higher level of lack of reliability perceived by the respondents on the overall healthcare system (72 out of 137) and diagnostic procedures (73 out of 137), followed by lack of reliability on hospital management (55), and non-availability of advanced treatment facilities at an affordable cost (54) (Figure 1). These findings were aligned with previous studies on the factors contributing to medical tourism, including user satisfaction about the quality of treatment facilities, costs of medical care, hospital management, and availability and skill levels of healthcare professionals [14,15].

A considerable number of respondents who had gone abroad shared their mistrust in the professional efficiency of doctors (33.57%) and healthcare staff (13.13%) (Figure 1). These observations might be considered in strategy formulation by policymakers and stakeholders while planning for organising specialised medical facilities at the local level, taking required initiatives to ensure the professional and interpersonal skill development of doctors, healthcare staff, and individuals involved in the management of hospitals, along with efficient, reliable diagnostic procedures.

In addition to the individuals utilising medical tourism in search for the healthcare of their families, more than half of the respondents (n = 285; 54.49%) revealed an intention to go out of the country for their treatment purposes (Figure 2), of whom an overwhelming proportion (n = 258; 90.52%) expressed their opinion in favour of the establishment of specialised hospitals in their localities which was also shared by their counterparts who did not intend to go abroad yet felt the necessity for specialised hospitals within their reach (n = 217 out 238; 91.17%) (Figure 2). Collectively, 475 out of 523 respondents (90.82%) explicitly reported the need for specialised hospitals accessible to them. Considering these findings, we can claim that a significant gap existed between specialised medical care available at regional-level facilities and the demand, despite the provisions of scarce specialised medical care at the national level over the years. This trend details the necessity of addressing the lack of adequate health facilities along with the associated dimensions of ensuring positive perceptions about healthcare facilities, supportive infrastructure, skilled workforce [16], and components of the health equality model, namely, empathy, tangibility, efficiency, and safety being pivotal for the overall satisfaction with the healthcare system of the country [17].

Division-wise need for specialised hospitals

Table 3 describes the frequency of demand for condition-specific specialised hospitals cited by the respondents from different regions of Bangladesh. The rows of the heatmap corresponded to the country’s primary administrative divisions, and the columns represented the demand for specialised facilities. The intensity of the colour, from lighter yellow to deep red, indicated the relative count of the responses in favour of a desired tertiary care facility, with higher counts depicted in darker shades. The data revealed a striking demand for hospitals specialised in medical care for cardiovascular diseases, diabetes, cancer, and renal diseases, irrespective of the localities of the inhabitants and a noticeable difference in demand for respiratory diseases (n = 42) and other health conditions (n = 34) by the respondents from the Rajshahi division (Figure 3). These findings also indicated a perceived need for specialised hospital care across the divisions.

This outline of demand is supported by the existing distribution of specialised care facilities heavily concentrated in the capital of Dhaka offering tertiary-level care for conditions, including national-level healthcare institutes dedicated to cardiovascular diseases, chest diseases, cancer, eye diseases, ear, nose, and throat diseases, gastroliver diseases, neurological diseases, and trauma and burn conditions, sparing only four specialised hospitals at the division level [2]. The data further highlighted specialised care for cardiovascular diseases, diabetes, cancer, renal diseases, and respiratory diseases as urgent health priorities across the surveyed regions, determining the necessity for targeted health policy interventions and resource allocation for specialised care facilities in areas with the highest reported needs.

Principal component analysis

The DBSCAN clustering analysis (Figure 4) identified multiple distinct groups within the survey respondents, revealing varied patterns of medical treatment preferences and demographic features. The visualisation of clusters after PCA dimensionality reduction showed clear separation of respondents into diverse clusters alongside numerous noise points, which DBSCAN classified as outliers. The analysis identified 12 clusters, alongside noise, with the most significant proportion (212 points) labelled as noise. These noise points were characterised by a moderate average age of 35 years, a foreign treatment preference rate of 55.7%, a high need for specialised hospitals at 79.7% (Figure 4) compared with the overall preference of 54.49% and demand of 90.82% for tertiary facilities, respectively (Figure 1), most common occupation as service holder, and 36.3% of individuals sought treatment abroad (Figure 4).

Analysing the clusters in detail revealed noteworthy differences across groups. For instance, Cluster 1 and Cluster 8 represented respondents who have a 100% preference for foreign treatment and a 100% need for specialised hospitals. However, Cluster 1 participants did not actually go abroad, whereas Cluster 8 did, with all members having service holders as the most common occupation. Clusters with younger average ages (Clusters 2, 3, and 10) consisted mainly of students or those categorised as others in occupation, all showed a universal need for specialised hospitals, but minimal preference for overseas treatment experience. Some clusters, such as Cluster 5 and Cluster 7, showed 100% actual overseas treatment, despite no foreign treatment preference recorded, highlighting possible complexities between preference and action. Most clusters exhibited a near-universal consensus on the need for specialised hospitals, elaborating a strong demand across demographic segments for enhanced requirements for setting up local healthcare facilities. This clustering approach thus demonstrated the heterogeneity of patient profiles yet distinct patterns regarding treatment decisions, which can guide tailored healthcare policies and targeted support for specific groups at regional levels.

Impact of the findings

Despite facing numerous challenges, including limited public health infrastructure, shortages of skilled healthcare workers, insufficient financial investment, and ongoing political instability, Bangladesh has made significant progress in achieving the health-related MDGs, particularly MDG 4 and MDG 5 [8]. While the private sector is expanding and mainly delivers tertiary healthcare services, the country still lacks a comprehensive national health policy to strengthen the system. The most pressing issue remains the absence of strong, proactive leadership capable of formulating and implementing effective policies to reinforce and advance the health system. With committed and visionary stewardship, Bangladesh could realise meaningful health sector reforms founded on the principles of equity and accountability, ultimately improving the nation’s overall health outcomes [18].

However, as Bangladesh lacks adequately reliable, scientific data on the dimensions of ever-growing trend in medical tourism further extended due to the rise in aging populations and increase in economical capabilities of larger population, our study could bring important directions to fill in the gaps in current policy formulation and strategical reformation of the healthcare system observed in earlier studies [19], enabling enhanced reliance on healthcare services by its own population to reduce the desire for medical tourism by providing state-of-the-art medical care accessible by the people living far from the capital. At the same time, the healthcare sector can collaborate with the Department of Tourism to promote a more favourable image of medical care in Bangladesh, both within the country and abroad [20]. Given Bangladesh’s cost advantage and the gradually increasing number of trained healthcare professionals, these strengths could be leveraged to position the country as a hub for medical services not only for its own citizens but also for neighbouring nations.

Strengths and limitations

This study has several limitations that need to be acknowledged. First, the primary data was collected through a structured questionnaire administered via social media, which may have excluded less digitally active populations such as rural communities and older individuals, thereby limiting reproducibility and representativeness. Second, the responses were self-reported, which may be subject to recall errors, misinterpretation of questions, or social desirability bias. Third, regional coverage was uneven, with some divisions underrepresented, making it difficult to generalise the findings to the national level. In addition, estimates of outbound medical treatment also varied across sources. Finally, the research design was cross-sectional, offering only a snapshot at a point in time without capturing evolving healthcare needs. Another notable limitation was the participation by the urban community due to their IT literacy and increased engagement in social media platforms, which prevented the study from comparing the findings with the rural counterparts of the respondents.

Despite these limitations, the study provided important insights into public perception of the existing healthcare system of the country, specifically insights into the reasons causing people to go abroad for life-saving medical care which may be considered by the relevant stakeholders to design an improved healthcare delivery system that allows patients to access quality treatment facilities in their reach and preventing from spending valuable foreign currency on health tourism. The study was also one of the earliest of its kind that revealed an important outlook on the necessity of the establishment of specialised hospitals at regional levels for better access to quality healthcare by people living outside the capital city.

Further studies

Future studies could be initiated involving a wider population with a randomised approach to evaluate the reality of outbound health tourism and further scope of disease-specific specialised hospitals based on the local demand at the regional level, considering the region-specific morbidity profiles and volume of the population.

## Conclusions

Medical tourism is not merely a treatment-seeking behaviour; rather, it is a tale of suffering experienced by individuals and families, taking a toll on their quality of life physically, psychologically, and financially. Establishing need-based specialised medical care facilities at divisional levels may reduce the burden of medical tourism of the Bangladeshi population, paving the way for strengthening the contemporary healthcare system. Moreover, initiating quality specialised treatment facilities may also alter the paradigm towards attracting potential groups of people from neighbouring countries in South Asia, Southeast Asia, and the Middle East. Nonetheless, it is anticipated that pertinent national policymaking groups, stakeholders, and development partners will act promptly to plan, allocate resources, and undertake the required actions to support tertiary-level healthcare at the regional level. Bangladesh can therefore extend a warm welcome to its neighbours, opening its doors to health tourism and boosting the country’s productivity and prosperity, supported by its globally renowned tourist attractions, internationally recognised heritage, and outstanding natural beauty.

## Appendices

Questionnaire for data collection

Study title: Need Assessment for Specialised Hospitals at a Regional Level: A Potential Way Out of Medical Tourism

Declaration: This survey is conducted solely for academic purposes. The results will be presented collectively, and no individual information will be disclosed. The privacy of respondents will be strictly maintained.

1. Age: ---------------- years.

2. Sex: □ Male □ Female □ Others □ Do not want to disclose.

3. Division:

□ Chattogram □ Dhaka □ Sylhet □ Mymensingh □ Khulna □ Rajshahi □ Rangpur □ Barishal

4. Educational status:

□ Up to primary

□ Up to secondary

□ Up to higher secondary

□ Up to Graduation

□ Postgraduation

5. Socioeconomic status:

□ Low (monthly household income: <12,500 Taka)

□ Middle (monthly household income: 12,501-21,000 Taka)

□ Upper (monthly household income: >21,000 Taka)

6. Occupation:

□ Student □ Housewife □ Service holder □ Business □ Others

7. Do you take any regular medication? □ Yes □ No

If yes, for which disease(s)? (You may select more than one.)

□ Diabetes

□ Hypertension

□ Renal diseases (kidney diseases)

□ Cardiovascular diseases (heart diseases, stroke)

□ Respiratory diseases, i.e., tuberculosis, asthma, COPD

□ Hepatobiliary diseases (liver/gallbladder diseases)

□ Cancers

□ Others (including infectious diseases, i.e., malaria, typhoid fever, etc.; neglected tropical diseases, i.e., filariasis, helminthiasis, etc.; other metabolic diseases, i.e., thyroid disorders)

8.   Do any of your family members receive treatment for any disease? (You may select more than one.)

□ Diabetes

□ Hypertension

□ Renal diseases (kidney diseases)

□ Cardiovascular diseases (heart diseases, stroke)

□ Respiratory diseases, i.e., tuberculosis, asthma, COPD

□ Hepatobiliary diseases (liver/gallbladder diseases)

□ Cancers

□ Others (including infectious diseases, i.e., malaria, typhoid fever, etc.; neglected tropical diseases, i.e., filariasis, helminthiasis, etc.; other metabolic diseases, i.e., thyroid disorders)

9. Did you or your family members go abroad for treatment?

□ Yes □ No

10. What are the reasons for going abroad for treatment? (You may select more than one.)

□ Lack of reliability on the overall healthcare system

□ Lack of reliability on diagnostic procedures

□ Lack of reliability in hospital management

□ Lack of reliability on doctors

□ Lack of reliability on healthcare staff

□ Non-availability of advanced treatment at a lower cost

11. Will you travel abroad for further treatment for you or your family members?

□ Yes □ No □ No comments

12. Do you think that it is necessary to establish a specialised hospital at the regional level?

□ Yes □ No □ No comments

13. If yes, what kind of specialised hospital should be established in your area?

□ Diabetes

□ Hypertension

□ Renal diseases (kidney diseases)

□ Cardiovascular diseases (heart diseases, stroke)

□ Respiratory diseases, i.e., tuberculosis, asthma, COPD

□ Hepatobiliary diseases (liver/gall bladder diseases)

□ Cancers

□ Others (including infectious diseases, i.e., malaria, typhoid fever, etc.; neglected tropical diseases, i.e., filariasis, helminthiasis, etc.; other metabolic diseases, i.e., thyroid disorders)

Date of data collection:

Name and Signature of the Data Collector:

Comment by the data collector:
